# Adeft: Acromine-based Disambiguation of Entities from Text with applications to the biomedical literature

**DOI:** 10.21105/joss.01708

**Published:** 2020-01-16

**Authors:** Albert Steppi, Benjamin M. Gyori, John A. Bachman

**Affiliations:** 1 Laboratory of Systems Pharmacology, Harvard Medical School

## Summary

For machines to extract useful information from scientific documents, they must be able to identify the entities referenced in the text. For example, in the phrase “binding of ligand to the IR is reduced”, “IR” refers to the insulin receptor, a gene with official symbol INSR. This process of identification, known as *named entity disambiguation* or *grounding*, requires the text string for the entity to be mapped to an identifier in a database or ontology. A complicating factor is that multiple distinct entities may be associated with the same text, leading to ambiguity. In scientific and technical documents, this ambiguity frequently originates from the use of overlapping acronyms or abbreviations: for example, in the biomedical literature, the term “IR” can refer not only to the insulin receptor, but also to ionizing radiation, ischemia reperfusion, insulin resistance, and other concepts. While interpreting these ambiguities is rarely a problem for human readers given the context of the whole document, it remains a challenge for text mining tools, many of which process text one sentence at a time.

Adeft (Acromine-based Disambiguation of Entities From Text) is a Python package for training and using statistical models to disambiguate named entities in text using document context. It is based on Acromine, a previously-published algorithm that assembles a training corpus for the different senses of an acronym by searching the text for defining patterns (DPs) (Okazaki & Ananiadou, 2006; Okazaki, Ananiadou, & Tsujii, 2010). Defining patterns typically take the form of parenthetical expressions, e.g. “long form (shortform)”, which can be identified systematically with regular expressions (for example, in the preceding sentence, “defining patterns (DPs)” is a defining pattern).

Disambiguation of abbreviations is a special case of word sense disambiguation (WSD) (McInnes & Stevenson, 2014; Navigli, 2009; Schuemie, Kors, & Mons, 2005). It is recognized as easier than disambiguation of general terms, first, because the existence of defining patterns allows for automatic labeling of text corpora, and second, because the senses of overlapping abbreviations tend to be more distinct than for general ambiguous terms (Stevenson & Guo, 2010; Stevenson, Guo, Al Amri, & Gaizauskas, 2009). Sophisticated methods have been developed for general WSD (Le, Postma, Urbani, & Vossen, 2018; Loureiro & Jorge, 2019; Luo, Liu, Xia, Chang, & Sui, 2018), but for the specific case of abbreviations, simple classification methods as used by Adeft achieve 98–99% prediction accuracy for most shortforms (Liu, Teller, & Friedman, 2004; Okazaki et al., 2010; Stevenson et al., 2009).

Given a named entity shortform (e.g., “IR”) and a set of texts containing the shortform, Adeft first uses the Acromine algorithm to identify candidate longforms (e.g., “insulin receptor”, “ionizing radiation”, etc.) by searching for defining patterns. Second, the user selects the subset of longforms relevant to their text mining use case and maps them to uniform identifiers either manually or programmatically (e.g., “insulin receptor” is mapped to gene symbol INSR, whereas “ionizing radiation” is mapped to MESH ID D011839). In addition to its Python API, Adeft provides a simple web-based interface to facilitate the curation of these mappings.

Third, Adeft stratifies the source documents according to the defining patterns they contain, resulting in a training corpus with multiple subsets of documents, one for each target concept (a concept may be associated with multiple longforms).

Based on this training corpus, Adeft builds logistic regression models (one for each entity shortform) that can be used to disambiguate an entity given the full text of the document. Adeft uses the Python package Scikit-learn (Pedregosa et al., 2011) to normalize the word frequencies for the documents in the training corpus by term frequency-inverse document frequency (TF-IDF), and then trains logistic regression models to predict the entity identity from the normalized word frequency vectors.

Once trained, these models can be used to disambiguate entities in new documents (including those not containing the defining pattern). Downstream applications make use of Adeft models by loading the appropriate model for the shortform and passing the enclosing text to the AdeftDisambiguator.disambiguate method. The method returns the top grounding along with a dictionary including probabilities for all alternative groundings. Adeft has already been integrated into the Integrated Network and Dynamical Reasoning Assembler (INDRA), a system that assembles mechanistic information from multiple natural language processing systems (Gyori et al., 2017). INDRA uses Adeft in its grounding_mapper submodule to re-ground ambiguous entities from external NLP systems.

In addition to the tools provided to build disambiguation models, Adeft also facilitates the use of pre-trained models for 46 ambiguous acronyms from the biomedical literature. However, the methods used by Adeft are not specific to any particular domain or type of document. In addition to documentation, the Adeft repository contains Jupyter notebooks demonstrating Adeft workflows, including the use of pre-trained models and the construction of new ones.
